# Conductive Bridge Random Access Memory (CBRAM): Challenges and Opportunities for Memory and Neuromorphic Computing Applications

**DOI:** 10.3390/mi13050725

**Published:** 2022-04-30

**Authors:** Haider Abbas, Jiayi Li, Diing Shenp Ang

**Affiliations:** School of Electrical and Electronic Engineering, Nanyang Technological University, Singapore 639798, Singapore; haider.abbas@ntu.edu.sg (H.A.); jiayi004@e.ntu.edu.sg (J.L.)

**Keywords:** CBRAM, RRAM, memristor, emerging memory technologies, neuromorphic computing, artificial synapses, artificial neurons

## Abstract

Due to a rapid increase in the amount of data, there is a huge demand for the development of new memory technologies as well as emerging computing systems for high-density memory storage and efficient computing. As the conventional transistor-based storage devices and computing systems are approaching their scaling and technical limits, extensive research on emerging technologies is becoming more and more important. Among other emerging technologies, CBRAM offers excellent opportunities for future memory and neuromorphic computing applications. The principles of the CBRAM are explored in depth in this review, including the materials and issues associated with various materials, as well as the basic switching mechanisms. Furthermore, the opportunities that CBRAMs provide for memory and brain-inspired neuromorphic computing applications, as well as the challenges that CBRAMs confront in those applications, are thoroughly discussed. The emulation of biological synapses and neurons using CBRAM devices fabricated with various switching materials and device engineering and material innovation approaches are examined in depth.

## 1. Introduction

Conventional memory technologies have been performing well for data storage applications over the last few decades. These famous existing memory technologies include dynamic random access memory (DRAM), static random access memory (SRAM) and flash memory. The DRAM and SRAM are volatile memories, whereas flash memory is a nonvolatile memory. All of these conventional memories rely on the storage of charges for the information storage. DRAM gives high memory density owing to its smaller cell size, which consists of a transistor and a capacitor (1T1C). However, the charge stored in the capacitor needs to be refreshed every few milliseconds, resulting in higher energy consumption. On the other hand, SRAM is fast, but it is not efficient for high-density memory applications owing to a larger memory cell containing six transistors (6T). Flash memory offers low-cost high-density nonvolatile memory. A flash memory cell consists of a floating-gate MOS transistor (FG-MOSFET). It utilizes the storage of the charges in the floating gate of the transistor for memory. A high voltage (>10 V) is needed to charge the floating gate, which gives higher energy consumption and low operation speed. Moreover, flash memory is almost at its physical and technological limits.

The inherent technical and physical scaling challenges of the conventional charge storage-based memory technologies prevent them from reaching nodes below 10 nm. These limitations have led to an increase in interest in developing new and next-generation memory technologies [1]. Various emerging memory technologies have been investigated in recent years, including phase-change memory (PCM), magnetoresistive random access memory (MRAM) and resistive random access memory (RRAM, ReRAM) [2,3,4]. The memory cells in these next-generation memories rely on the change in resistance of switching materials to store information rather than the charge storage. Among the emerging memory technologies, RRAM has been widely investigated owing to its promising switching behaviors. RRAM is one of the most promising candidates for next-generation memory applications. RRAM comprises a fast switching speed associated with SRAM, high storage density comparable to that of DRAM, and nonvolatility associated with flash memory, making it very attractive as an alternative to current memory technologies.

Furthermore, in the big data era, a rapid increase in the amount of data is apparent and requires fast and efficient processing. The conventional computing systems are convenient to use for general-purpose tasks, but they are not efficient for the emerging data-intensive jobs. The CMOS-based conventional computing systems are inefficient due to the low density of on-chip memories and the von Neumann bottleneck. Conventional computing systems are based on the von Neumann architecture in which memory and computing units are physically separated; this leads to time and energy consumption as data is constantly transferred between the two. In recent years, neuromorphic computing has emerged as an alternative to conventional computing systems owing to its brain-inspired features such as in-memory-computing, adaptive learning abilities and massive parallelism [5,6]. For the realization of a neuromorphic computing system, emerging electronic devices, including RRAM, have shown excellent features in emulating the essential functions of the biological brain [5].

**Figure 1:**
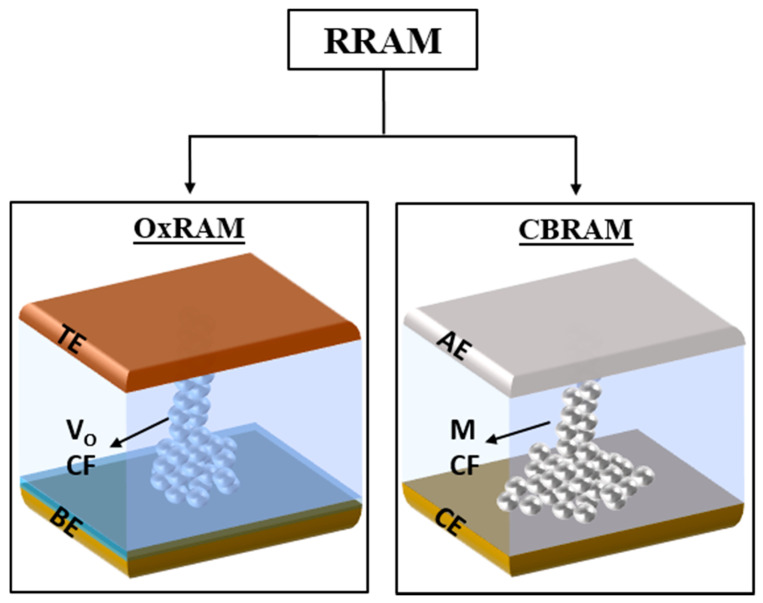
A schematic representation of the classification of RRAM on the basis of switching mechanisms as: OxRAM and CBRAM.

Generally, RRAM is also called a ‘memristor’, short for memory resistor, or resistive switching memory (RSM). RRAM offers easy fabrication and high-density memory owing to a simple metal-insulator-metal (MIM) structure. Each memory cell compromises a switching material (insulator/semiconductor) sandwiched between two metal electrodes [7,8]. Due to the simple structure, it also offers the best scalability features. The resistance of the switching material is varied between a high resistance state (HRS) and a low resistance state (LRS) in a controlled manner to store data. Interestingly, multilevel cell (MLC) storage capability can also be achieved in RRAM by programing multiple resistance states between HRS and LRS for multibit storage applications [9,10]. Based on the basic switching mechanisms, RRAM is classified into two types: metal oxide random access memory (OxRAM) and conductive bridge random access memory (CBRAM), as shown in Figure 1. The OxRAM is also called valance change memory (VCM), whereas the CBRAM is also called electrochemical metallization memory (ECM) [11]. In the OxRAM cell, the switching layer is always a metal oxide. The metal oxide layer is sandwiched between the two electrodes, which are called the top electrode (TE) and the bottom electrode (BE). The major switching mechanism in OxRAM involves the formation and rupture of the oxygen vacancy (V_O_)-based conductive filament (CF) in the oxide switching layer [12]. On the other hand, the switching layer in the CBRAM cell can be any solid electrolyte. The electrolyte switching layer is sandwiched between an electrochemically active electrode (AE) and an inert counter electrode (CE). The details about different materials that are used as the AE, CE and the electrolyte switching layer are discussed in Section 2. The switching mechanism in CBRAM involves the migration of metal ions (M+) from the AE forming a metallic (M) CF between the AE and CE, as shown in Figure 1. When a positive voltage is applied to the AE, an electrochemical reaction occurs, oxidizing the AE metal into cations (M+) which drift through the solid electrolyte switching layer under the electric field and are reduced at the CE. The CF, which is composed of the metal atoms of the AE, connects the CE and AE like a conductive bridge switching the device to LRS. Electrochemical dissolution of the metallic CF occurs when the voltage polarity is reversed, resetting the device to HRS. CBRAM devices have been extensively studied in both academia and industry for memory and neuromorphic applications owing to their simple and easily controllable switching mechanism. In 2012, CBRAM became the first commercially available RRAM [13,14]. CBRAM offers excellent switching performance such as fast switching, low power consumption, very low operating voltage (<1 V), MLC storage, etc. Moreover, CBRAM exhibits versatile switching characteristics, including volatile threshold switching (TS) and nonvolatile memory switching properties (MS), which broaden its application areas to volatile memory, nonvolatile memory, and selector device for memory and emulation of biological synapses and neurons for neuromorphic applications. The CBRAM with volatile TS characteristics is also called a ‘diffusive memristor’, which recently attracted great attention for its applications in emerging memories and neuromorphic computing areas [15,16]. Although CBRAM offers excellent opportunities for memory and neuromorphic applications, it also suffers some challenges such as poor retention, low endurance, highly stochastic switching, etc.

In this review, the opportunities that CBRAM devices offer for volatile and nonvolatile memory, as well as neuromorphic computing systems, have been discussed along with the challenges they face for these applications. The electrode and switching materials used for the fabrication of CBRAM devices are discussed first, including their pros and cons for different applications. The switching mechanisms involved in CBRAM are reviewed in detail. Various recent studies on CBRAM devices for memory applications are discussed, including the different methods utilized for improvement of variability and retention and endurance characteristics. We then discuss the emulation of biological synapses and neurons for neuromorphic applications with CBRAM devices fabricated using various materials, including conventional and emerging materials.

## 2. Electrode and Switching Materials

**Figure 2:**
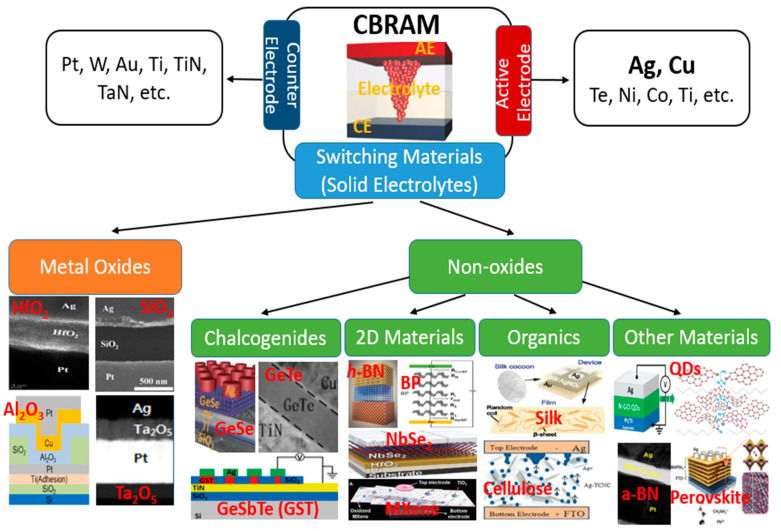
The schematic representation of a CBRAM cell with a detailed classification of the materials that are used for CE, AE and switching medium in a CBRAM device. The switching materials are classified into two categories: metal oxides [17,18,19,20] and non-oxides. The non-oxides are further classified as chalcogenides [21,22,23], 2D materials [24,25,26,27], organic materials [28,29] and other emerging materials [30,31,32].

As we mentioned in the introduction, the CBRAM cell has a two-terminal MIM structure where an electrolyte switching layer (I) is sandwiched between two metal electrodes (M). In a CBRAM cell, one of the two metal electrodes should be an electrochemically active metal, which is called the AE, whereas the other electrode consists of an inert metal, which is called the CE. Usually, the AE is deposited on the switching layer as a TE; however, it is not necessary to always deposit the AE as the TE. The variety of materials that are used in a CBRAM device for CE, AE and the electrolyte switching layer are schematically presented in Figure 2.

For the CE, any conductive material can be used as long as it is inert and does not diffuse easily into the switching layer. The most commonly used materials for the CE in CBRAM devices include Pt, W, Au, Ti, TiN, TaN, etc. Ag and Cu are the most commonly used electrochemically active metals that are used as the AE in CBRAM devices owing to their high diffusivity in the switching layer [33,34]. However, other metals have also been utilized as the AE, such as Te, Ni, Co, Ti, etc. [19,24,35,36,37]. Nonetheless, most of the studies conducted on CBRAM devices used Ag or Cu as the AE. Moreover, it is possible for some metals to behave as both inert and active electrodes. It is an interesting property of the CBRAM that any material can be utilized as the switching layer as long as the switching medium allows the diffusion of metal ions of the AE through it. As a result, CBRAM devices can be fabricated using a wider range of materials for the switching layer. Metal oxides are the most widely used materials in RRAM devices due to their easy fabrication and compatibility with the existing CMOS devices. Therefore, metal oxides have been extensively used in the fabrication of CBRAM devices as well. Here, we have categorized the switching materials used in CBRAM devices into two categories: metal oxides and non-oxides, as shown in Figure 2. Several transition metal oxides are utilized as the switching media in CBRAM devices. Among the non-oxide switching materials, a diverse range of materials, including chalcogenides, two-dimensional (2D) materials, organic materials and various other emerging materials, are adopted as solid electrolytes.

### 2.1. Electrode Materials

For the material to be used as the CE in a CBRAM cell, there is no stringent requirement as it simply acts as an inert electrode. Usually, Pt or W is used as the CE material. However, the selection of AE material is critical as it is the material that differentiates between OxRAM and CBRAM. Various switching characteristics are affected by the choice of different AEs. Moreover, the thickness of the AE layer also affects the switching mechanisms in CBRAMs [38]. Several active metals have been utilized as the AEs in various CBRAM devices. Moreover, the transparent CBRAM devices with a conducting oxide (ITO) as the AE showed diffusion of indium from ITO into the SiCN switching layer, denoting that the selection of AE material is not limited to the electrochemically active metals, but a broader range of AEs can be explored by properly selecting the switching layer and the AE material [39].

**Figure 3:**
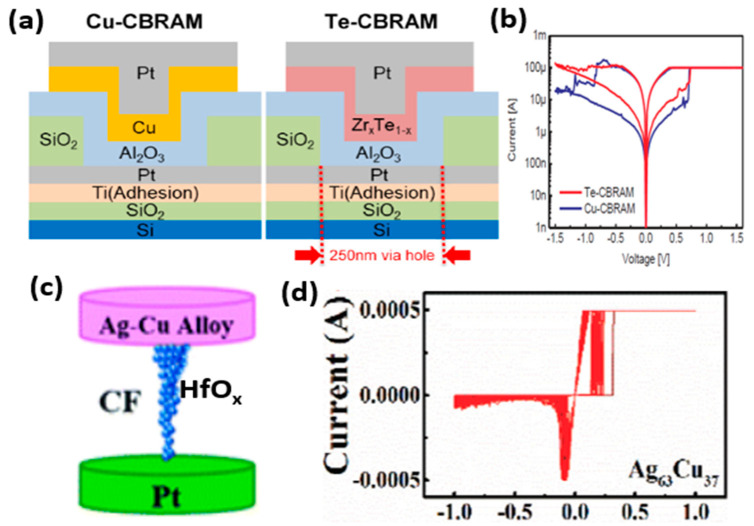
The CBRAM devices with different AE materials. (**a**) The schematic diagrams of the Cu-CBRAM and Te-CBRAM devices. (**b**) The basic I-V characteristics of the Cu-CBRAM and Te-CBRAM devices. Reprinted from Ref. [19]. (**c**) The schematic diagram of the CBRAM device with Ag-Cu alloy-based AE. (**d**) The I-V characteristics of the Ag–Cu alloy-based CBRAM device with an Ag-Cu ratio of 63:37. Reprinted from Ref. [40].

Ag and Cu are the most commonly used metals as AEs. Due to their higher diffusivity, the Ag and Cu-based CBRAM devices typically exhibit low-voltage operation leading to low power consumption. Moreover, the higher ionic mobility of Ag and Cu ions provides faster switching speeds. Various other active metals have also been used as AEs in CBRAMs, including metal alloys. Lee et al. compared the resistive switching properties of the Al_2_O_3_ CBRAM devices with Cu and Te-based AEs (Figure 3a) [19]. For the AE, Zr_x_Te_1−x_ layers of various compositions are deposited by a co-sputtering of Zr and Te. Both Cu and Te-based devices showed bipolar resistive switching, as shown in Figure 3b. The Te-based device presented better retention properties at higher temperatures due to the formation of a relatively large CF. Instead of only Ag or Cu metals, their alloys used as the AE. In a recent study, the HfO_x_-based CBRAM devices, utilizing Ag–Cu alloy as the AE, showed a strong dependence of the switching characteristics on the alloy electrode ratios (Figure 3c) [40]. The electrode alloying is employed in this study to improve the switching irregularities in CBRAM devices, particularly improving the variabilities in the SET voltage distributions. The devices with an Ag–Cu ratio of 63:37 presented better uniformity in the distribution of the switching voltages with faster switching speeds and low power consumption (Figure 3d). The electrode alloying gives better control of the source, position and electrochemical activity of the CFs.

### 2.2. Switching Materials

For the switching layer (solid electrolyte) in the CBRAM devices, many different materials can be used as long as the material can transport metal cations through it effectively. As a result, a broader range of materials can be used for the fabrication of CBRAM devices. As the range of switching materials is too broad, we have classified them as metal oxides and non-oxides for the discussion here.

#### 2.2.1. Metal Oxides

Among various materials that are used in CBRAMs, metal oxides have attracted greater interest owing to their excellent switching capabilities, easy fabrication and compatibility with the existing semiconductor fabrication processes. Metal oxides are among the most widely investigated materials for RRAM devices, particularly for OxRAM [41,42]. For the CBRAMs, the insulating metal oxides can be utilized as solid electrolytes by depositing the oxide thin films of a few nanometers in thickness. A stronger electric field in the extremely thin metal oxide films and a shorter travel distance for the metal ions support the ionic conduction through the insulating oxide thin films. Generally, CBRAM suffers poor retention compared to OxRAM, which is critical for nonvolatile memory applications. However, the selection of metal oxides as the switching layer ensures improved retention compared to the other solid electrolytes.

**Figure 4:**
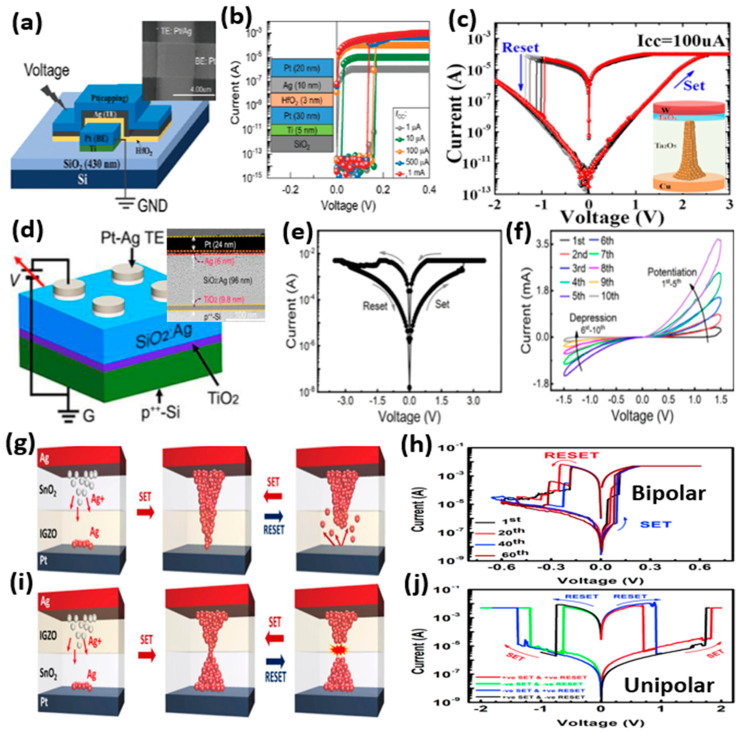
The CBRAM devices with metal oxide-based switching materials. (**a**) The schematic diagram of the crossbar structure of the Ag/HfO_2_/Pt CBRAM device with a top-view SEM image of the cross-point. (**b**) The I–V characteristics of the Ag/HfO_2_/Pt device under different compliance currents present TS characteristics. Reprinted from Ref. [33]. (**c**) The typical I-V characteristics of the CBRAM device with a bilayer Ta_2_O_5_/TaO_x_ switching medium. The inset shows a schematic of the device structure. Reprinted from Ref. [43]. (**d**) The schematic diagram of a CBRAM device based on SiO_2_:Ag and TiO_2_ bilayer switching medium with a cross-sectional TEM image of the device. The I-V characteristics of the SiO_2_:Ag and TiO_2_ bilayer-based device present (**e**) digital bipolar switching and (**f**) and bidirectional analog switching. Reprinted from Ref. [44]. (**g**) The schematic diagram of the device with a switching layer stacking of SnO_2_/IGZO and (**h**) its corresponding I–V characteristics with bipolar switching. (**i**) The schematic diagram of the device with a switching layer stacking of IGZO/SnO_2_ and (**j**) its corresponding I–V characteristics with unipolar switching. Reprinted from Ref. [45].

A large number of metal oxides have been investigated as the switching layer for the CBRAM devices, such as HfO_2_, SiO_2_, Ta_2_O_5_, Al_2_O_3_, TiO_2_, etc. [17,18,19,20]. Various thin film properties, including the thickness and the composition of the switching layer, greatly affect the switching characteristics of the CBRAM. Keeping in view the high scalability attributes of the CBRAM, it is always desirable to fabricate very thin switching layers. In a recent study, Chekol et al. fabricated a 3 nm HfO_2_-based CBRAM device with a device structure Ag/HfO_2_/Pt (Figure 4a) [33]. Figure 4b shows the typical I–V characteristics of the CBRAM device, which displays TS characteristics under different values of compliance current. The occurrence of a volatile TS and nonvolatile MS is easily controlled on CBRAMs by controlling the size of the CF. The formation of a weaker CF leads to volatile TS, whereas a stronger CF yields MS. The weak CF in the diffusive memristors with TS undergoes a self-rupture procedure and the self-rupture time (relaxation time) is dependent on the size and morphology of the CF. This relaxation time of the CF is very critical because by controlling the relaxation time of the filament in CBRAM, a variety of applications can be realized, including memory, artificial synapses and neurons and hardware security applications.

Moreover, multilayer metal oxides are used as the switching materials in the CBRAMs to enhance the switching characteristics of the devices. Yu et al. used a tantalum oxide homogeneous bilayer switching medium with a different stoichiometry for each layer [43]. The CBRAM device with a Ta_2_O_5_/TaO_x_ bilayer stack presents highly reliable and uniform bipolar switching (Figure 4c). The Ta_2_O_5_ layer acts as the main switching medium whereas the nonstoichiometric thin layer of TaO_x_ acts as an external resistance suppressing the current overshoot during the SET process, presenting a self-compliance switching. Moreover, a CBRAM device fabricated with a bilayer oxide switching medium with Ag-doped SiO_2_ (SiO_2_:Ag) and TiO_2_ presented bipolar switching with better endurance characteristics (Figure 4d,e) [44]. The SiO_2_:Ag/TiO_2_ bilayer oxide structure enables a gradual Ag-filament growth and rupture in the switching medium. The gradual CF formation and rupture provide the bidirectional analog switching in the SiO_2_:Ag/TiO_2_ CBRAM device, which is vital for emulation of the biological synapses (Figure 4f). This type of bidirectional analog switching is usually difficult to attain in CBRAM devices, due to abrupt switching, which hinders their applications for synaptic emulation.

Furthermore, it is interesting to note that diverse switching behaviors can be observed in CBRAMs by engineering the stacking sequences of oxide thin films in a bilayer switching medium. The CBRAM devices fabricated with bilayers of SnO_2_ and IGZO with different stacking showed contrasting switching behaviors in both oxide stacks [45]. The CBRAM device with the switching layer of SnO_2_/IGZO presented bipolar switching characteristics (Figure 4g,h), whereas its reverse stack IGZO/SnO_2_ showed unipolar switching (Figure 4i,j). This behavior is attributed to the formation of CFs with different shapes in both devices owing to the difference in the diffusion rates of Ag ions in the SnO_2_ and IGZO switching layers (Figure 4g,i). Both bipolar switching and unipolar switching offer opportunities depending on their applications. Metal oxides are among the most widely used switching materials in CBRAMs not only because they offer excellent switching properties with easy and cost-effective fabrication processes, but they also offer opportunities to deal with the challenges that CBRAMs encounter with comparatively simple yet efficient techniques.

#### 2.2.2. Non-Oxides

As we mentioned above, literally any material can work as the switching material in CBRAMs as long as it allows the metal ions from the AE to diffuse into and drift through it, thus forming metallic CFs. Hence, it is challenging to categorize all of the non-oxide solid electrolytes into different groups. Here, we have classified the non-oxide switching materials as chalcogenides, 2D materials, organic materials and other emerging materials (Figure 2). A number of chalcogenides have been utilized as the solid electrolytes in CBRAMs such as GeSe, GeS, GeTe, etc. [21,22,23]. Moreover, research into CBRAMs based on 2D materials has been sparked by recent advances in 2D materials [46]. The CBRMs based on 2D *h*-BN and black phosphorus (BP) presented excellent resistive switching characteristics [25,47,48]. Furthermore, the utilization of organic materials for electronic devices is a step toward the realization of green electronics for minimizing the electronic waste that is being generated by traditional electronic materials [49]. The CBRAMs fabricated with biodegradable organic materials, such as silk, cellulose, chitosan, etc., show promising switching characteristics, along with their environmentally friendly nature, for sustainable research and development [28,29]. Moreover, other emerging materials also show promising switching characteristics when used as the switching materials in CBRAMs, such as quantum dots (QDs), amorphous boron nitride (a-BN), perovskites, etc. Sokolov et al. presented repeatable volatile TS characteristics in the nitrogen-doped graphene oxide quantum dots (GOQDs)-based CBRAM devices, which were utilized for the emulation of essential synaptic functions [30].

**Figure 5:**
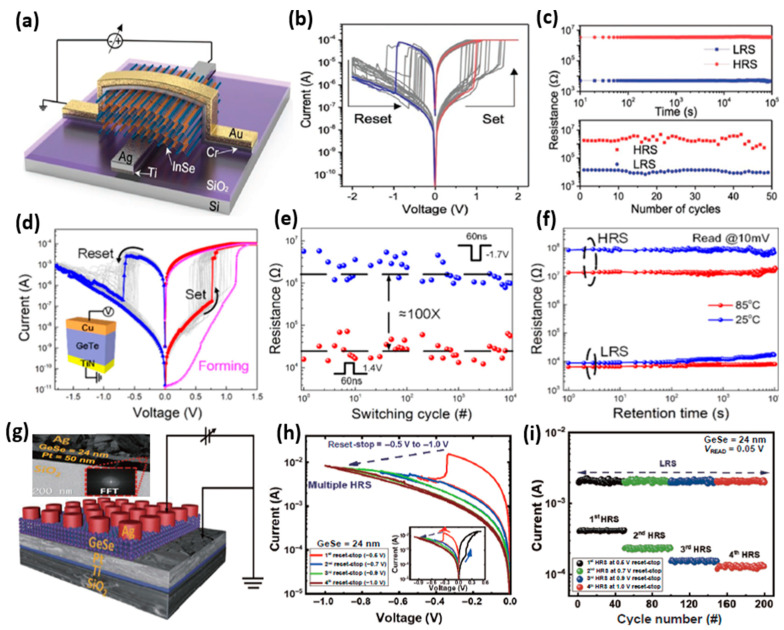
The CBRAM devices with chalcogenide-based switching materials. (**a**) The schematic diagram of a CBRAM device with InSe 2D monochalcogenide switching layer, (**b**) its typical I–V characteristics presenting bipolar switching, and (**c**) its retention and endurance characteristics. Reprinted from Ref. [50]. (**d**) The typical I–V characteristics of a CBRAM device with structure Cu/GeTe/TiN presenting bipolar switching. The inset shows the schematic of the device structure. (**e**) The endurance characteristics of the Cu/GeTe/TiN device. (**f**) The retention characteristics of the Cu/GeTe/TiN device. Reprinted from Ref. [21]. (**g**) The schematic diagram of a CBRAM device with a monochalcogenide GeSe switching layer and a cross-sectional TEM image of the device. (**h**) The I–V characteristics of the 24 nm GeSe-based device presenting multilevel switching and (**i**) the endurance characteristics of the multiple resistance states. Reprinted from Ref. [23].

Among the several non-oxide switching materials that are used in CBRAMs, chalcogenides are the most favorable electrolyte materials owing to their high ionic conductivities. In fact, chalcogenides are the most widely used and investigated electrolyte materials for CBRAMs. Chalcogenides are good solid electrolytes that offer superior switching properties such as faster speeds and low voltage switching. Both amorphous chalcogenides, as well as layered 2D transition-metal dichalcogenides (TMDs), offer excellent switching characteristics for CBRAM applications. Furthermore, 2D TMDs offer precise control of the metallic filament growth in the thin layered switching medium due to which an incremental growth and rupture of filaments can be achieved, thereby leading to analog switching which is desirable for synaptic emulation. The bandgap of the TMDs can also be varied depending on the metal-chalcogen composition, which provides more options to tune the properties of TMDs-based devices. The layered devices are fabricated by exfoliating monolayer and multilayer TMDs. The monolayer TMDs provide possibilities for fabricating ultrathin switching layers, which is beneficial for device scaling as well as for very low-power applications. Mazumder et al. reported bipolar switching in a CBRAM device fabricated with a layered InSe 2D monochalcogenide switching layer (Figure 5a,b) [50]. As shown in Figure 5c, the thin (8.8 nm) layered switching material-based device presented nonvolatile switching with a stable retention time of >10^5^ s, maintaining an ON/OFF ratio of >10^3^. In another study, Jin et al. fabricated CBRAMs with a 35 nm amorphous chalcogenide GeTe-based solid electrolyte [21]. The CBRAM devices with a structure of Cu/GeTe/TiN presented bipolar switching with a low forming voltage (Figure 5d). Excellent switching characteristics such as better endurance with faster switching speed (60 ns) and stable retention at high temperatures are demonstrated (Figure 5e,f). In a recent study on amorphous monochalcogenide, GeSe-based CBRAM devices presented different switching behaviors depending on the thickness of the amorphous monochalcogenide solid electrolyte [23]. The Ag/GeSe/Pt devices are fabricated by varying the thickness of the GeSe switching layer from ~6 nm to ~56 nm (Figure 5g). The devices with thicker and thinner GeSe layers showed unipolar and bipolar switching, respectively. The CBRAM device with a 24 nm GeSe switching layer presented a stable multilevel switching (Figure 5h,i). The multilevel switching is beneficial for multibit memory applications and it is essential for emulation of the synaptic functions of the biological synapses to realize artificial synapses for neuromorphic applications.

## 3. Switching Mechanisms

The resistive switching in CBRAMs involves the formation and rupture of metallic CFs in the electrolyte switching layer tuning the resistance of the electrolyte between an HRS and LRS. For the bipolar switching devices, the metal cations from the AE diffuse into the switching layer under a positive bias, which upon meeting the electrons injected from the CE are reduced to metal atoms. The aggregation of metal atoms inside the switching layer forms the CF, which connects the CE and the AE. This switches the device from HRS to LRS, turning it ON. This process is also called the SET process. The device undergoes a RESET process with a negative biasing of the AE. For the negative voltage polarity, the metallic filament undergoes an electrochemical dissolution process which creates a disconnection between the electrodes, switching the device back to HRS. For the unipolar switching devices, the mechanism of the SET process is similar to that of the bipolar switching; however, the reset mechanism is different. This is because the unipolar switching does not depend on the voltage polarity; instead, it is voltage amplitude-dependent. After the formation of CF, the filament can be ruptured at either positive or negative voltage. When high currents flow through the CF, it is ruptured at its weakest point due to Joule heating. Moreover, the CBRAM devices, usually, require high voltage to undergo the first SET process from their pristine state. This initial switching from the pristine HRS to LRS is called electroforming. During the high voltage forming process, a large number of metal ions diffuse into the pristine switching layer, forming a metallic CF which upon RESET does not fully rupture. During the subsequent SET process, a lower SET voltage reconnects the CF in the ruptured part. The fabrication of forming-free devices is highly desirable to avoid this undesirable forming process.

It is important to note that the number of CFs and the morphology and shape of the CF that is formed during the SET process are not the same in all CBRAM devices. It depends on several factors, including the ionic conductivities of the switching media, the diffusion rates of various AE materials, the thickness of the switching medium, etc. Moreover, the morphology and shape of the CF are also dependent on the device operation such as the magnitude of the applied voltage and current compliance imposed during the SET and electroforming processes. The shape and morphology of the CF are momentous in CBRAMs as the existence of different types of switching behaviors greatly depends on the morphologies of the filaments formed. Depending on the morphology, shape and strength of the CFs, volatile TS and nonvolatile MS can be observed in CBRAMs. The formation of a smaller and weaker CF leads to TS, whereas a strong filament or multiple CFs induce MS behavior. The switching behavior can also be controlled by tuning the CF morphologies during device operation. The possibility of controlling CF morphologies during the device operation provides opportunities to CBRAMs for versatile applications. Moreover, the shape of the CF also determines the possibility of the existence of unipolar or bipolar switching [45].

**Figure 6:**
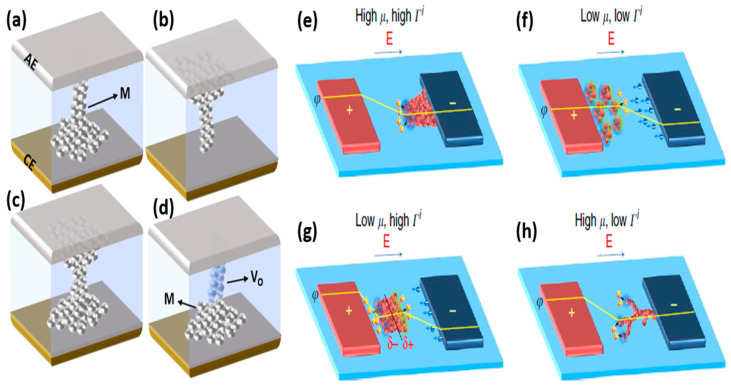
Formation of the CFs in CBRAMs with different shapes and compositions as (**a**) inverted conical shape, (**b**) conical shape, (**c**) hourglass shape and (**d**) hybrid CF composed of metal atoms and oxygen vacancies. Qualitative model showing CF growth dynamics governed by kinetic parameters such ad ion mobility (μ) and redox rates (Γ^i^). When (**e**) both μ and Γ^i^ are high, (**f**) both μ and Γ^i^ are low, (**g**) μ is low but Γ^i^ is high, and (**h**) μ is high but Γ^i^ is low. Reprinted from Ref. [34].

Various shapes of the filaments have been proposed by different research groups. In most of the studies, an inverted cone-shaped CF formation is proposed which is thicker at the CE interface and thinner near AE (Figure 6a). This type of CF starts growing at the surface of the CE where the AE cations are reduced to metal atoms after interacting with the electrons from CE. The CF grows from the CE towards AE, giving it an inverted conical shape. Another possibility is the formation of a conical filament with a thicker part near AE and thinner at CE (Figure 6b). The conical and inverted cone-shaped CFs are usually reported in bipolar switching devices. Moreover, filaments with the shape of an hourglass are also proposed, where the thinnest part of the filament lies at the center of the CF (Figure 6c). The position of thinner and thicker parts of the CF is important for the operation of CBRAMs as the thinnest part of the CF undergoes rupture and reformation during the successive switching. The devices with this type of CF usually present unipolar switching, where the CF is easily ruptured at the center (weak point) by the joule heating. In addition to the pure metallic CFs, hybrid filaments are also observed in CBRAMs (Figure 6d). Lee et al. proposed the formation of a hybrid CF, which is composed of Ag atoms and oxygen vacancies (V_O_), in Ta_2_O_5_-based CBRAM devices [18]. The composition of the CF was controlled by the compliance current, where at a lower compliance current, V_O_-dominated CF was observed, whereas a hybrid CF was formed at the higher compliance current.

Furthermore, the growth dynamics of CFs in the CBRAMs greatly depend on kinetic factors such as ion mobility (μ) and redox rates (Γ^i^) [34]. For higher μ and Γ^i^, the CF starts forming rapidly at the CE and grows towards the AE, leading to an inverted conical CF (Figure 6e). This is because the metal ions can quickly reach the CE, thereby avoiding the formation of metallic nanoclusters in the switching layer due to higher μ and Γ^i^, whereas when both μ and Γ^i^ are low, the CF starts growing near AE (Figure 6f). Metal islands are formed inside the switching layer and CF formation occurs through cluster migration. The CF grows from AE towards the CE. For low μ and high Γ^i^, nucleation is experienced away from the AE and CE but near the middle of the solid electrolyte, as shown in (Figure 6g). The filament further grows between the AE and the cluster filament formed in the middle, and then it starts growing towards the CE. In contrast, for high μ and low Γ^i^, the AE metal ions reach the CE rapidly due to a high μ and filament starts growing at the CE (Figure 6h). However, a lower supply of metal cations due to the low Γ^i^ leads to the formation of branched CFs in this case.

**Figure 7:**
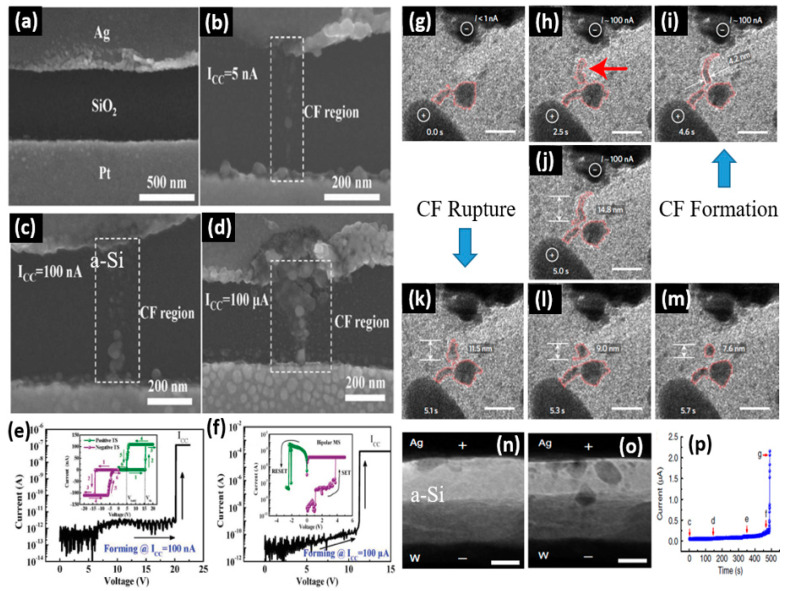
The visualization of formation and rupture of metallic CFs in CBRAMs. (**a**) SEM image of an Ag/SiO_2_/Pt device before the electric bias is applied. The SEM images of the device presenting the morphologies of the CFs at compliance currents of (**b**) 5 nA, (**c**) 100 nA and (**d**) 100 μA. The corresponding I-V characteristics of the electroforming and subsequent switching at compliance currents of (**e**) 100 nA presenting volatile TS and (**f**) 100 μA presenting nonvolatile MS. Reprinted from Ref. [20] In situ TEM images of an Au/SiO_x_N_y_:Ag/Au device showing the dynamics of CF growth and rupture. (**g**–**j**) The time sequence of formation of the CF. (**k**–**m**) The time sequence of the spontaneous self-rupture of the CF. Reprinted from Ref. [51] The TEM images of an Ag/a-Si/W device recorded (**n**) before forming and (**o**) after forming. (**p**) The *I*–*t* characteristics of the forming process. Reprinted from Ref. [52].

The formation and rupture of metallic CFs in CBRAMs have been experimentally demonstrated using high-resolution electron microscopy techniques by several research groups. Sun et al. demonstrated pseudo-bridging CFs in a planar CBRAM device with a structure Ag/SiO_2_/Pt (Figure 7a) [20]. The CF growth for the forming processes under different compliance currents was analyzed via scanning electron microscopy (SEM). The morphologies of the CFs formed at compliance currents of 5 nA, 100 nA and 100 μA are shown in Figure 7b–d, respectively. For lower compliance currents (5 nA and 100 nA), the formation of weak and discontinuous CFs consisting of isolated Ag nanoparticles are observed, resulting in a volatile TS behavior (Figure 7b,c), whereas for higher compliance current (100 μA) forming, the CF grows stronger and becomes continuous without any gap between the Ag nanoparticles, leading to nonvolatile MS (Figure 7d). The corresponding I–V characteristics of forming and subsequent switching at compliance currents of 100 nA and 100 μA are shown in Figure 7e,f, respectively, presenting the volatile TS and nonvolatile MS behaviors. In another study, Wang et al. investigated the metallic CF formation and its spontaneous dissolution behavior in a planar Au/SiO_x_N_y_:Ag/Au device using in situ high-resolution transmission electron microscopy (HRTEM) [51]. The time sequence of HRTEM images in Figure 7g–i show the formation of the filament, whereas Figure 7k–m shows the self-rupture behavior of the CF. A constant 20 V is applied for 5 s under a compliance current of 100 nA. After a delay of ~2 s, the Ag nanoparticles started growing in the gap between Au electrodes (Figure 7g–i). At 5 s, the formation of a CF having a length of 14.8 nm is observed (Figure 7j). After 5 s, the power was turned off to observe the relaxation of the CF. It can be noted that the Ag CF rapidly contracted to 11.5 nm at 5.1 s, which finally reduced to a circular profile having a diameter of 7.6 nm (Figure 7k–m). Yang et al. demonstrated CF formation in a vertical CBRAM device with structure Ag/a-Si/W using in-situ TEM [52]. The real-time growth of the CF was directly observed during the forming process on a pristine device, as shown in Figure 7n–p. The *I*–*t* characteristics during the forming process are shown in Figure 7p and the TEM images recorded before and after forming, corresponding to the points c and g in Figure 7p, are shown in Figure 7n,o, respectively. The filament grows from the AE towards the CE, with a thicker filament near the AE region forming a canonical shape Ag CF (Figure 7o).

## 4. Memory Applications

The transistor-based conventional memory technologies have demonstrated huge progress over the years for volatile and nonvolatile memory applications. For the current data storage needs, they are fulfilling the requirements to some extent. However, recently, a rapid increase in the amount of data that is generated every day has been noticed which is expected to further increase in the coming years, owing to larger growth in the IT industry. As conventional devices are approaching their scaling limits, new emerging memory technologies are being investigated to fulfill future data storage needs efficiently. Among the various emerging memory technologies, CBRAM demonstrates better technical features, making it one of the most favorable candidates for future memory applications. Although CBRAM offers better opportunities for future data storage, it also faces challenges in its practical applications. Intensive research has been carried out in recent years to find solutions to challenges and to explore the vast opportunities that CBRAM offers for memory applications. A summary of the key memory characteristics of the CBRAM devices based on different electrode and switching materials is presented in Table 1.

### 4.1. Volatile Memory

**Figure 8:**
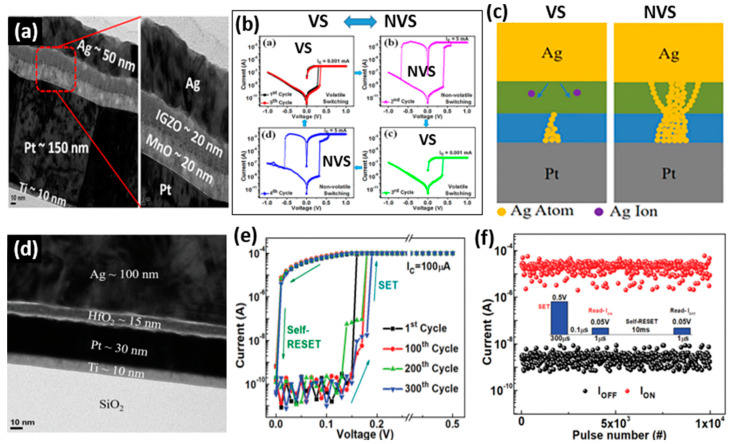
(**a**) The cross-sectional TEM image of a CBRRAM device with an IGZO/MnO bilayer switching medium. (**b**) The I-V characteristics of the bilayer device presenting a reversible transition between volatile switching and nonvolatile switching. (**c**) The schematic of the switching mechanisms during both volatile and nonvolatile switching. Reprinted from Ref. [57]. (**d**) The cross-sectional TEM image of an Ag/HfO_2_/Pt CBRRAM device. (**e**) The typical I-V characteristics of the Ag/HfO_2_/Pt device presenting highly uniform volatile TS and (**f**) its pulse endurance characteristics for the volatile TS. Reprinted from Ref. [17].

Generally, nonvolatile memory is preferred over volatile memory, owing to the high power consumption in volatile memories. However, for specific applications, volatile memories are also needed. As mentioned in the previous section, the morphology and size of the CFs in CBRAM devices can be controlled in such a way that the switching can be tuned as volatile or nonvolatile switching. The existence of volatile and nonvolatile switching can be controlled during the device operation. Among other factors, the thickness of the switching layer is also critical for defining the device switching behavior. For both volatile and nonvolatile memory applications, CBRAM offers excellent switching with fast switching speeds and low-voltage operation. Moreover, the retention time of the volatile CBRAMs can be tuned from a few milliseconds up to several minutes, which can be beneficial for data security applications where data retention time can be pre-programmed. The data will be automatically erased after the programmed retention time. In a recent study, the CBRAM devices with a bilayer metal oxide switching layer Ag/IGZO/MnO/Pt presented a coexistence of volatile and nonvolatile switching characteristics (Figure 8a) [57]. Interestingly, the volatile and nonvolatile switching modes showed a reversible transition behavior (Figure 8b). Generally, the transition from volatile to nonvolatile switching is not reversible owing to thicker CF formation during nonvolatile switching which cannot undergo a self-rupture process. However, the Ag/IGZO/MnO/Pt device showed the coexistence of volatile and nonvolatile switching with a reversible transition behavior, confirming that the device can be utilized for volatile as well as nonvolatile memory applications. The SET process, at a low compliance current, limits the CF to the MnO layer, providing volatile switching, whereas switching at a higher compliance current induces a stronger filament in the bilayer along with multiple smaller CFs in the IGZO layer, leading to a nonvolatile switching (Figure 8c). Similarly, an HfO_2_-based CBRAM with a simple device structure of Ag/HfO_2_/Pt also presented volatile switching behavior (Figure 8d) [17]. The device presents reliable unidirectional volatile switching for the device operation at a compliance current of 100 µA (Figure 8e). The very low-voltage switching (~0.2 V) guarantees power-efficient device operation. The endurance characteristics for the volatile switching with a self-RESET are shown in Figure 8f. The device exhibits repeatable endurance for 10^4^ switching cycles without any failure.

### 4.2. Nonvolatile Memory

Every electronic device requires nonvolatile memory storage components for its operation. The capacity of the storage varies depending on the application of the device. As we have seen enormous growth in the IT industry in recent years, the need for high-capacity efficient nonvolatile memory devices has increased further. CBRAM offers great opportunities to fulfill the demands of future nonvolatile memory storage, owing to its efficient switching properties and the high-scalability features of the CBRAM cells. Although CBRAM devices exhibit excellent prospects for future memory applications, they also face challenges that need to be overcome. The device engineering and material innovation techniques offer efficient solutions to the challenges CBRAMs face. CBRAMs often undergo an unwanted negative-SET behavior that degrades device reliability and switching uniformity. Wu et al. utilized interface engineering in the ZrO_2_-based CBRAM device by inserting a 2D MoS_2_ layer at the CE interface [54]. The engineered device with a structure of Ag/ZrO_2_/MoS_2_/Pt eliminated the negative-SET behavior, leading to greatly improved endurance and uniform switching.

**Figure 9:**
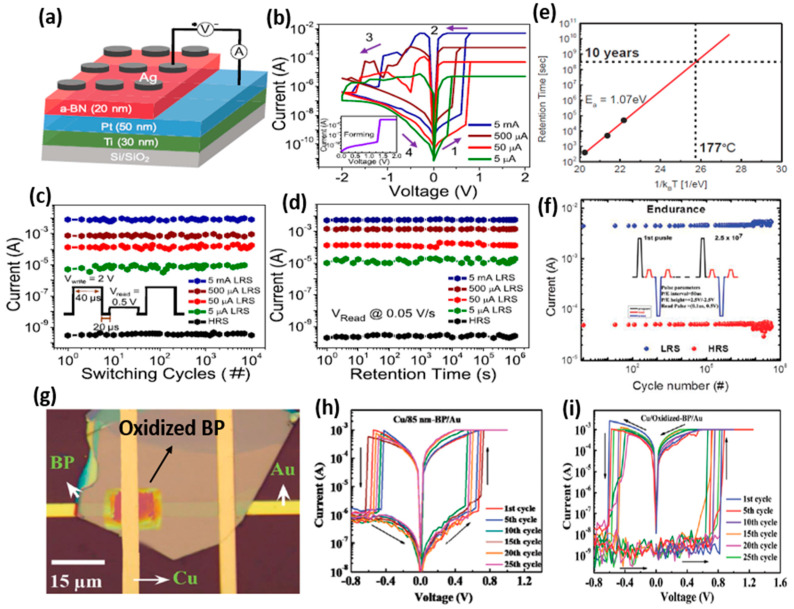
(**a**) The schematic diagram of an a-BN-based CBRAM device. (**b**) The typical I–V characteristics of the Ag/a-BN/Pt device under different compliance currents presenting multilevel switching characteristics. (**c**) The highly stable pulse endurance and (**d**) retention characteristics of the multiple resistance states demonstrating the feasibility of the device for multibit data storage applications. Reprinted from Ref. [58]. (**e**) The data retention time of 10 years at 177 °C was demonstrated by an Al_2_O_3_-based CBRAM device with Te as the AE material. Reprinted from Ref. [19]. (**f**) A higher switching endurance of >10^7^ cycles was presented by the engineered r-TiO_2_-based CBRAM device. Reprinted from Ref. [61]. (**g**) The optical image of a BP-based device. (**h**) The I-V characteristics of the Cu/BP/Au CBRAM device and (**i**) Cu/Oxidized-BP/Au device. Reprinted from Ref. [25].

In a recent study, Khot et al. demonstrated high-density memory applications of the CBRAMs in a-BN-based devices (Figure 9a) [58]. The Ag/a-BN/Pt device presents multilevel switching achieved by controlling the compliance current during the SET process, as shown in Figure 9b. The multilevel switching confirms the multibit data storage abilities of the CBRAMs, which is highly desirable for designing high-density memory storage devices. However, for practical multibit applications, the reliability of multilevel switching is very important. The Ag/a-BN/Pt device demonstrates highly repeatable and reliable multilevel states with a uniform resistance distribution during the repeated switching up to 10^4^ cycles (Figure 9c). Moreover, each resistance state presents memory retention characteristics of over 10^6^ s, confirming its applicability for nonvolatile memory applications (Figure 9d). Typically, a memory device should demonstrate a retention time of over 10 years at a higher temperature for its application as a nonvolatile memory device. However, CBRAM shows poor retention compared to other emerging memories. Lee et al. compared the retention characteristics in Al_2_O_3_-based CBRAM devices with Cu and Te as the AE material [19]. The Te-based device exhibited better data stability at high temperatures, providing a data retention time of 10 years at 177 °C (Figure 9e). The demonstration of a higher retention time was attributed to the formation of wide effective CF in the Te-based CBRAM. This indicates that the size of the CF mainly affects the data retention characteristics of the CBRAM devices. Another challenge that CBRAM faces is the high randomness in the filament formation and rupture, which often leads to poor endurance characteristics. To address this issue, a number of approaches have been implemented. One of the most efficient approaches is to limit the formation of the CF to a specific predefined path in the switching layer [59,60]. Abbas et al. used a solution-processed rutile TiO_2_ (r-TiO_2_) to create the predefined channels in the r-TiO_2_ switching layer [61]. This approach was successful in suppressing the high stochasticity in the CF formation, presenting a higher switching endurance of >10^7^ cycles (Figure 9f). Moreover, the material innovation offers effective solutions for the fabrication of reliable CBRAM devices. Several new materials have been investigated recently for their possible applications as the switching media in the CBRAMs. Rehman et al. used an emerging 2D layered material, BP, as the switching layer in Cu/BP/Au CBRAM devices (Figure 9g) [25]. As the BP films are highly vulnerable to degradation on exposure to ambient conditions, the native oxide was grown on BP devices as a protective layer using deep ultraviolet light. The CBRAM devices with oxidized and non-oxidized BP switching layers presented highly reliable nonvolatile bipolar switching, as shown in Figure 9h,i, respectively. The device with an environmentally stable oxidized-BP layer exhibited an improved ON/OFF ratio of 10^5^, which is advantageous for nonvolatile memory applications.

### 4.3. Selector for Memory

**Figure 10:**
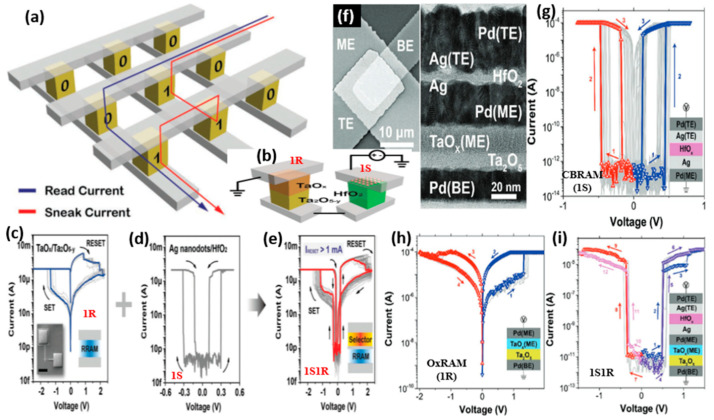
The application of the CBRAM as a selector device. (**a**) The schematic diagram of a crossbar array presenting the concept of sneak path current. (**b**) The schematic diagram of a series combination of a bilayer OxRAM and a CBRAM as the memory (1R) and selector (1S) devices. The corresponding I-V characteristics of the individual (**c**) 1R element with bipolar switching, (**d**) 1S element with bidirectional TS and (**e**) combined 1S1R combination presenting a successful implementation of the selector with memory. Reprinted from Ref. [69]. (**f**) The cross-sectional and top-view images of a 1S1R device fabricated using a CBRAM device as a selector and an OxRAM as a memory element. The typical I–V characteristics of the (**g**) 1S element, (**h**) 1R element and (**i**) the combined 1S1R device. Reprinted from Ref. [70].

Among various emerging memory technologies, RRAM is capable of offering extremely high-density memories owing to its high-scalability features, which are the result of its simple two-terminal structure. For high-density memories, fabrication of large crossbar arrays is needed. However, memristor crossbar arrays are subject to leakage currents, which pose a significant barrier to their wider application [62,63]. The selected cross-point cell in an array suffers from a sneak path current from neighboring cells during write and read operations. The sneak current adds to the actual read current, which reduces the read accuracy and increases the power consumption (Figure 10a). This can also affect the computation speed in neuromorphic computing applications. The sneak path currents become more severe when the size of the crossbar arrays is increased. This issue is fundamental and hinders the broad deployment of memristor crossbar arrays. Therefore, to minimize the effects of the sneak current, the RRAM device should be engineered to have a highly non-linear I–V relation. The devices with complementary or self-rectifying switching characteristics show non-linear I–V suppressing the sneak current [64,65,66]. However, engineering the device switching in this manner is a difficult challenge. Another method is to add a selector device in series with the RRAM device at each crossbar cell. A diode or transistor can be used as a selector device. Traditional selectors, such as transistors, however, hinder the scalability of memristor crossbar arrays due to the large feature size of the transistor. Herein, the CBRAM provides effective opportunities to mitigate the sneak path problem because of its volatile TS properties with low OFF current and high ON/OFF ratio [67,68]. The sneak path currents in crossbar arrays can be effectively suppressed by using a CBRAM device with a volatile TS (also known as diffusive memristor or threshold switch) as a selector (1S) in series with an RRAM device (1R) in a one-selector-one-resistor (1S1R) configuration (Figure 10b).

Song et al. demonstrated a CBRAM threshold selector with a structure Ag/TiO_2_/Pt presenting high selectivity (~10^7^) and steep slope (<5 mV/decade) [71]. Furthermore, this Ag/TiO_2_-based device was utilized as a selector (1S) with another Cu/Ge_2_Sb_2_Te_5_-based CBRAM (1R) forming a 1S1R configuration for crossbar array applications [56]. The 1S1R configuration presented low leakage current, uniform resistance distribution and stable retention. Hua et al. used a series combination of OxRAM and CBRAM devices as the memory (1R) and selector (1S) devices, respectively (Figure 10b) [69]. The I-V characteristics of the individual bilayer OxRAM device and the CBRAM threshold selector device are shown in Figure 10c,d, respectively. For the AE of the selector device, Ag nanodots were used instead of Ag thin film. This provided stable CF growth by minimizing the excessive Ag migration from the AE into the solid electrolyte. The threshold selector device presented a forming free switching with a large ON current, high ON/OFF ratio (>10^9^) and steep switching slope (0.65 mV/decade). The I-V characteristics of the combined 1S1R device are presented in Figure 10e. With a high endurance, the 1S1R device exhibits significant suppression of the leakage current, confirming its suitability for crossbar array applications. In another study, Midya et al. combined the CBRAM and OxRAM devices as a selector and RRAM, respectively, to fabricate the 1SIR devices (Figure 10f) [70]. The individual CBRAM device (1S) with a structure Pd/Ag/HfO_x_/Ag/Pd showed highly repeatable bidirectional TS with an extremely high selectivity of 10^10^ (Figure 10g). The bilayer OxRAM device (1R) having a structure Pd/Ta_2_O_5_/TaO_x_/Pd presented bipolar switching characteristics (Figure 10h). The vertically integrated 1S1R device presented excellent selection characteristics for applications in large crossbar arrays for nonvolatile memory and neuromorphic computing (Figure 10i).

## 5. Neuromorphic Computing Applications

The conventional computing systems, which we use today, are much more efficient for general-purpose tasks. These systems are based on a von Neumann architecture, where the computing system consists of separate computing and memory units. There is a physical separation between the two most important units. This physical separation between the memory and processing units, known as the so-called memory wall, gives high power consumption and slow processing speeds, thus limiting the efficiency of conventional computing systems. Therefore, the development of a new computing system is needed for the efficient computation of emerging data-intensive tasks. As an alternative to traditional computing, neuromorphic computing, which is inspired by the operation of a biological brain, has emerged in recent years [15]. Neuromorphic computing combines memory and processing as the biological brain, offering in-memory-computing opportunities. A fundamental and essential step toward realizing hardware-based neuromorphic computing systems is emulating the functions of biological synapses and neurons. Several conventional and emerging devices have been investigated to mimic biological synapses and neurons. A large feature size, however, prevents conventional devices from providing scalability capabilities. CBRAM is one emerging device that offers excellent prospects for achieving artificial neurons and synapses [72].

### 5.1. Emulation of Biological Synapses

**Figure 11:**
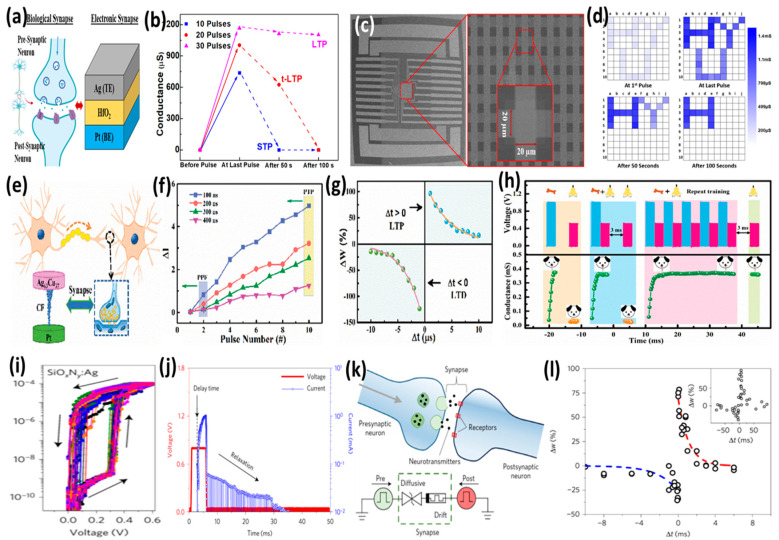
Emulation of essential biological synaptic functions with CBRAM devices. (**a**) The schematic images of the biological synapses and HfO_2_ CBRAM-based artificial electronic synapses. (**b**) Demonstration of the transition of STP to LTP depending on the pulse stimulations in Ag/HfO_2_/Pt CBRAM devices. (**c**) Top-view SEM image of the Ag/HfO_2_/Pt crossbar array device. (**d**) Image memorization into the crossbar array demonstrating the STM and LTM behaviors of the brain with a transition from STM to LTM for higher memorization rehearsals. Reprinted from Ref. [17]. (**e**) A schematic representation of implementing an artificial synapse with a CBRAM device using Ag-Cu alloy as AE. The successful emulation of (**f**) SRDP and (**g**) STDP behaviors of the synapses in Ag_63_Cu_37_/HfO_2_/Pt device. (**h**) Experimental demonstration of Pavlov’s dog experiment. Reprinted from Ref. [40]. (**i**) The I-V characteristics of SiO_x_N_y_:Ag-based diffusive memristors presenting highly repeatable TS. (**j**) The delay, SET and self-relaxation behavior of the diffusive memristor. (**k**) The combination of the diffusive memristor with a drift memristor for emulation of synaptic functions. (**l**) The successful emulation of the STDP learning rules by the combined diffusive and drift memristors. Reprinted from Ref. [51].

There are roughly 10^11^ neurons and 10^15^ synapses in the human brain, which are considered to be the basic computation units. Synapses are the connections between neurons. Learning and memory functions of the brain are strongly influenced by the strength of the connections between neurons, i.e., the synaptic weight. As a result of activities in both presynaptic and postsynaptic neurons, synaptic strength is modified. This is widely known as synaptic plasticity [73,74,75]. In addition to mimicking the plasticity of synapses, the two-terminal structure of CBRAM is also very similar to the structure of biological synapses. The presynaptic and postsynaptic terminals in a biological synapse correspond to the AE and CE in CBRAM, while the synaptic cleft corresponds to the electrolyte switching layer (Figure 11a). Although the overall functionality of the biological system is very complex, the ionic drift of the K^+^ or Ca^2+^ ions in the biological synapses that modulate the synaptic weight has a correlation with the diffusion and drift of the metal ions (Ag^+^ or Cu^2+^) in the electrolyte layer that tune the conductance of the CBRAM device.

CBRAMs can mimic both short-term and long-term synaptic plasticity functions of the biological synapses by utilizing their volatile and nonvolatile switching characteristics. The short-term plasticity (STP) and long-term plasticity (LTP) behaviors of synapses were successfully emulated in HfO_2_-based CBRAM devices (Figure 11b) [17]. When the presynaptic terminal (AE) was fired with pulse trains having a different number of pulses in each train, varying conductance enhancement was observed depending on each pulse train. For the smaller number of pulses (10 pulses), the conductance could increase to a certain level, which showed further enhancement for the higher number of pulses (20 and 30 pulses). The device conductance was read just after each pulse train and at 50 and 100 s intervals to monitor the retention of the increased conductance state. It is interesting to note that, for the pulse train with 10 pulses, the device could not maintain the state for 50 s, confirming its short-term behavior, whereas for 20 pulses, the increased conductance state could be sustained for a while but eventually, it dropped at 100 s. This shows transient LTP (t-LTP) behavior. However, for 30 pulse stimulations, the enhanced conductance state could be sustained for a longer time with a smaller degradation, confirming its transition from STP to LTP. This behavior is attributed to the strength of the CF formed during the pulse stimulations with a different number of pulses. The smaller pulse stimulations form a weak CF that undergoes self-rupture in a short time, leading to STP behavior, whereas stronger stimulations with a higher number of pulses grow stronger and more robust CF, which does not experience self-rupture, instead sustaining for a longer time. The CBRAM devices are fabricated as crossbar arrays to implement image memorization for emulation of the short-term memory (STM) and long-term memory (LTM) behaviors of the brain (Figure 11c). Three different letters (‘H’, ‘Y’ and ‘U’) are stored in the crossbar array device using a different number of pulses for each letter. The letters (‘Y’ and ‘U’) programmed into the crossbar array using weaker stimulations (20 and 10 pulses) were stored in STM, whereas the letter (‘H’) programmed using stronger input stimulations (30 pulses) was stored in LTM (Figure 11d). In a recent study, Wang et al. emulated essential synaptic plasticity functions in the HfO_x_-based CBRAM devices fabricated with Ag–Cu alloy AEs (Figure 11e) [40]. The synaptic emulation is implemented on the device with an Ag–Cu ratio of 63:37, which presented better switching characteristics. The spike-rate-dependent plasticity (SRDP) behavior of the biological synapses is emulated by varying the inter-spike interval of the stimulating pulses (Figure 11f). Spike-timing-dependent plasticity (STDP) is one of the most important Hebbian learning rules of biological synapses. Therefore, it is essential for an artificial synapse to emulate STDP behavior for its application as an electronic synaptic device. CBRAMs have shown the ability to successfully emulate STDP behaviors of the synapses. The CBRAM device with Ag–Cu alloy AE could successfully implement the STDP learning rule, confirming its feasibility as an electronic synaptic device for neuromorphic applications (Figure 11g). Furthermore, the CBRAM can also imitate associative learning, which is one of the most complex learning processes occurring in the brain. One of the most well-known examples of associative learning is Pavlov’s dog experiment, which is often simulated by using artificial synapses [76,77]. Pavlov’s dog experiment is experimentally replicated on the Ag_63_Cu_37_/HfO_x_/Pt CBRAM device, demonstrating that it can imitate associative learning with two excitatory stimulation modes (Figure 11h).

Diffusive memristors with volatile TS possess excellent potential in brain-inspired computing because of their similarities with the ion transport mechanisms in biological neural systems. Wang et al. demonstrated highly repeatable TS in the SiO_x_N_y_:Ag-based diffusive memristors (Figure 11i) [51]. Under an applied pulse, the current jumped abruptly to LRS after a short delay and slowly increased further under bias (Figure 11j). The current then relaxed back to HRS in a specific relaxation time, as the voltage pulse ended. The delay and relaxation times showed a dependence on various factors, including the input voltage pulse parameters, which shows the feasibility of using diffusive memristors to tune the desired dynamics for neuromorphic systems. Moreover, the diffusive memristor was combined in series with a drift memristor (OxRAM) to demonstrate SRDP and STDP (Figure 11k). The combination resembles a synapse between presynaptic and postsynaptic neurons. The STDP behavior of the biological synapses was successfully emulated with a time difference between pre- and postsynaptic spikes applied to diffusive and drift memristors (Figure 11l).

The volatile TS characteristics of the CBRAM devices have been widely investigated for the emulation of biological synapses confirming the opportunities CBRAMs offer for the realization of neuromorphic computing. However, the CBRAMs generally present abrupt SET and RESET behaviors, which hinders the emulation of incremental weight modulation behaviors of the biological synapses. Analog switching with a gradual SET and RESET is highly desirable for the demonstration of long-term potentiation (LTP) and long-term depression (LTD) characteristics of the synapses [78]. OxRAMs offer great opportunities to implement highly linear LTP/LTD, owing to their easily attainable analog switching characteristics [41,79]. However, several device engineering and material innovation techniques have been explored to realize analog-type switching in CBRAMs as well.

**Figure 12:**
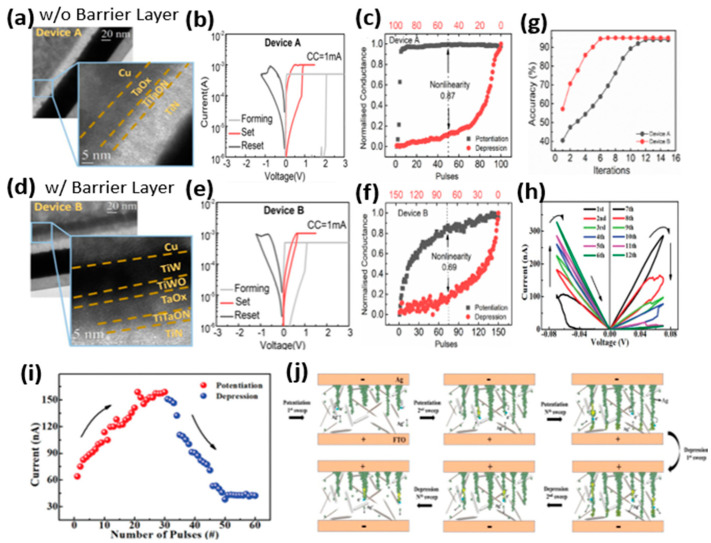
Analog switching in CBRAM for the synaptic emulation. (**a**) The cross-sectional TEM image of a CBRAM device fabricated without a barrier layer and (**b**) its corresponding I–V characteristics showing an abrupt switching behavior and (**c**) the LTP/LTD characteristics with a larger nonlinearity. (**d**) The cross-sectional TEM image of the engineered CBRAM device fabricated with a TiW barrier layer at the AE interface and (**e**) its corresponding I–V characteristics showing a gradual switching behavior and (**f**) the LTP/LTD characteristics with an improved nonlinearity. (**g**) Neural network training accuracy for devices with and without a barrier layer. Reprinted from Ref. [80]. (**h**) The I–V characteristics of an Ag nanoparticles-doped CNC-based CBRAM device showing analog switching and (**i**) its LTP/LTD characteristics. (**j**) The schematic illustration of the analog switching mechanism. Reprinted from Ref. [29].

Saleem et al. demonstrated a transformation of digital switching to analog switching in TaO_x_-based CBRAM devices by inserting an oxidizable metal diffusion barrier between the AE and switching layer [80]. The device without an additional barrier layer showed typical abrupt switching behavior (Figure 12a,b). This abrupt switching leads to a high nonlinearity in the LTP/LTD characteristics (Figure 12c). Moreover, a higher forming voltage was also needed to initiate the switching in the single-layer device. The engineered device was fabricated by adding a TiW barrier layer between the Cu AE and the TaO_x_ switching layer (Figure 12d). The additionally added barrier layer performed multiple functions. First, the excessive diffusion of Cu ions into the switching layer was limited by the barrier layer, suppressing the formation of multiple random CFs and leading to increased switching stability. Secondly, an interfacial layer of TiWO is formed at the interface of the switching layer and oxidizable barrier layer, which modulates the oxygen vacancy distribution at the top interface. This gives rise to the formation of a hybrid CF composed of both metal ions and oxygen vacancies. As a result, the abrupt switching transforms into a gradual switching (Figure 12e). Due to the repeatable analog switching characteristics, the engineered device presented a better emulation of LTP and LTD behaviors with improved linearity, which is a critical aspect of neuromorphic computing (Figure 12f). The linearity and symmetry in LTP and LTD are essential to achieving high recognition accuracy in hardware artificial neural networks (ANNs). The engineered analog CBRAM device performed faster recognition, achieving 95% accuracy with smaller interactions on a simulated neural network, as shown in Figure 12g. Moreover, new material innovations also provide opportunities to achieve analog switching in CBRAMs. A CBRAM device based on Ag nanoparticles doped cellulose nanocrystal (CNC) as the switching layer was capable of demonstrating extremely low-voltage analog switching (Figure 12h) [29]. The device successfully emulated several essential synaptic functions, including LTP and LTD, with better linearity (Figure 12i). The analog switching was achieved by keeping the operating voltages very low for modulating the conductance of the device in HRS. Multiple CFs were formed by the Ag nanoparticles doped into the CNC (Figure 12j). The conductance of the device was incrementally modulated by decreasing/increasing the gap between the CFs and the CE during LTP/LTD.

### 5.2. Emulation of Biological Neurons

**Figure 13:**
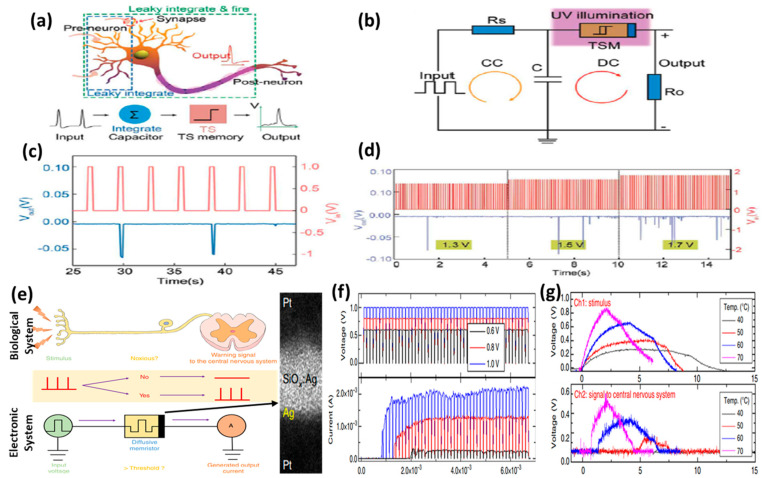
Emulation of biological neurons with CBRAM devices. (**a**) Schematic illustration of LIF mechanism in a biological neuron and artificial neuron. (**b**) Schematic of LIF neuron circuit. (**c**) The output signal of the LIF neuron circuit corresponding to successive input pulses. (**d**) The output spikes under voltage pulses with different pulse amplitudes. Reprinted from Ref. [82]. (**e**) Schematic illustration of the mechanisms of a biological nociceptor and an artificial nociceptor implemented with a diffusive memristor. A cross-sectional TEM image of the diffusive memristor is on the right. (**f**) Response of the device to multiple pulses with different amplitudes. (**g**) The generated voltage from the thermoelectric module for different temperatures and the response of the device to each voltage presenting a successful implementation of a thermal nociceptor. Reprinted from Ref. [84].

For the realization of a brain-like neuromorphic computing system, the emulation of biological neurons is as essential as that of the synapses. Memristor devices with volatile switching present efficient solutions for implementing artificial neurons [16,81]. The leaky integrate and fire (LIF) model, with a parallel combination of a leaky resistor and a capacitor, is utilized to describe biological neurons (Figure 13a,b). Because of their highly controllable volatile switching characteristics, diffusive memristors are ideal for simulating biological neurons. The threshold and self-relaxation properties of volatile CBRAMs are highly analogous to the functions of the biological neurons. The volatile nature of the CF with self-forgetting behavior mimics the leaky membrane potential of neurons. Zhao et al. emulated the functions of the biological neurons in silk protein-based volatile threshold switching CBRAM devices [28]. An LIF artificial neuron is realized based on the volatile switching characteristics of the devices. Applying a series of voltage pulses (100 Hz, 1 V) successfully triggers the threshold-driven firing behaviors of biological neurons. By changing the amplitude of applied pulses, the magnitude of the pre-neuronal stimulation is simulated where the spike frequency increases with increasing input pulse amplitude. A relevant feature is that the spike amplitude of the output neuron is about 0.1 V, which is close to the biological action potential.

Wang et al. reported the InP/ZnS quantum dots-based, optically modulated CBRAM devices with volatile and nonvolatile switching characteristics [82]. A transition from nonvolatile switching to volatile TS was demonstrated under UV illumination. A visual neuron-based LIF model was implemented as shown in Figure 13b. There are two loops in the LIF circuit: charging circle (CC) and discharging circle (DC). The memristor device is connected in series with an output resistor in the DC of the circuit and both components are in parallel connection with the capacitor. The memristor is kept under UV illumination to induce volatile TS. The input pulses are applied to the CC that charges the capacitor. During the charging of the capacitor, the voltage across its terminals continuously increases (integration) until it reaches the threshold voltage of the memristor. At the threshold voltage, the memristor switches to LRS and the capacitor is discharged (fire) through DC. During discharging, the memristor switches back to HRS as the voltage across it drops below the hold voltage. With a refractory period, integration and fire procedures repeat for multiple input voltage pulses (Figure 13c). The response of the output signal to different input voltage amplitudes is shown in Figure 6d. In a recent study, Zhao et al. realized an LIF artificial neuron using the volatile switching characteristics of silk-based CBRAM devices [28]. Moreover, Zhang et al. demonstrated artificial neurons with an ultralow-power consumption in a CBRAM device having a structure Pt/FeO_x_/Ag [83]. The artificial neuron was implemented on a single device, minimizing the need for auxiliary circuits, which provides opportunities for high-scale integration.

Furthermore, the volatile CBRAMs can also emulate nociceptors, which are specialized sensory neurons that are more sensitive to noxious stimuli. Nociceptors sense noxious stimuli and alert the brain to begin the motor response. Yoon et al. experimentally demonstrated an artificial nociceptor based on a CBRAM device with bidirectional volatile TS [84]. The schematic diagrams demonstrating the concept of a biological nociceptor and an artificial nociceptor with a correlation between both are presented in Figure 13e. The cross-sectional TEM image of the diffusive memristor used for the experimental demonstration of an artificial electronic nociceptor is also shown in the figure. The response of the device for pulse stimulation with different pulse amplitudes is presented in Figure 13f. The initial increase in the current clearly depends on the amplitude of the pulses. For the pulses with higher amplitudes, the initial current jump occurred after a short interval compared to that for the lower amplitude pulses. Utilizing these properties of the device, an artificial thermal nociceptor was demonstrated by connecting a thermoelectric module to the memristor. The voltage generated by the thermal module when heated at different temperatures and the corresponding response of the device are shown in Figure 13g. For the lowest temperature (40 °C), the systems did not generate any output alarm signal, indicating that no noxious stimulus is detected. For the higher temperatures, output alarm signals were generated depending on the temperature where the quickest and strongest signal was generated for the highest temperature (70 °C), indicating the presence of a noxious stimulus.

## 6. Summary and Outlook

In this review, the CBRAM devices for memory and neuromorphic computing applications are discussed. CBRAM offers great opportunities for memory and neuromorphic applications owing to its excellent switching attributes, including low-voltage operation, fast switching, high ON/OFF ratio, co-existence of volatile and nonvolatile switching characteristics, great scalability possibilities with a simple structure, etc. However, together with the outstanding opportunities that CBRAM offers for emerging applications, it also encounters several technical challenges, such as other emerging technologies, for its practical applications. To mitigate the challenges faced by the CBRAM devices, structural engineering and material innovation techniques have been utilized by different research groups. Developing devices that can meet the requirements of emerging memory and neuromorphic applications is still a difficult task. Therefore, further extensive research is needed to deal with the challenges and achieve better switching attributes in a single device. For the emulation of biological synapses to build a neuromorphic computing system, conductance linearity with longer retention and a larger ratio is very challenging to achieve. Controlling the metal ion migration is one of the key approaches to enhancing the linearity of conductance update during LTP/LTD in CBRAMs. Furthermore, by the formation of hybrid filaments and controlling the concentration of oxygen vacancies and metal ions in the switching layer, it is possible to improve the weight update linearity with larger intermediate conductance states.

For the fabrication of CBRAM devices, several materials have been utilized for AE, CE and the electrolyte switching layer. Unlike OxRAM, the AE is one of the most critical components in a CBRAM cell. The switching characteristics mainly depend on the selection of the AE and the switching material, whereas the CE usually just acts as an inert electrode and does not play an effective role in switching. Ag and Cu are the most commonly used AE materials. For the electrolyte switching layer, a large number of materials, ranging from conventional materials to exciting emerging materials, have been utilized. Considering the fact that CBRAMs face various challenges in their applications, research into engineering the switching materials may help resolve these issues, as well as expand the application range of CBRAMs. Moreover, further research on new materials also provides exciting opportunities for the CBRAMs. However, these emerging materials are not compatible with the existing CMOS technology. It is therefore necessary to conduct further research to understand the switching mechanisms of these materials and fabricate devices compatible with current manufacturing technology in order to find practical applications for them.

For memory applications, CBRAMs offer excellent opportunities owing to their versatile switching behaviors. For both volatile and nonvolatile memory applications, CBRAMs offer great switching characteristics, such as extremely fast operation, low power consumption, high ON/OFF ratio, etc. Additionally, volatile CBRAMs also show impressive capabilities as selector devices for suppressing sneak path currents in large crossbar arrays. Compared to OxRAM, CBRAM suffers from poor retention, which is challenging for its applications in nonvolatile storage. Despite the fact that different material engineering techniques have been used to improve the retention properties of the CBRAMs, further research is needed.

Emulating the functions of biological synapses and neurons is a crucial step in realizing hardware-based neuromorphic computing systems. For the emulation of synapses, CBRAMs offer opportunities to mimic both short-term and long-term synaptic plasticity functions owing to their volatile and nonvolatile switching characteristics. It is hard to achieve gradual switching in CBRAM devices, which is essential for mimicking the LTP and LTD behaviors of the synapses. Generally, CBRAM devices present an abrupt switching behavior, which is generally not very feasible for synaptic emulation. However, pulse engineering methods are utilized to precisely control the CF’s relaxation time in the volatile switching devices. By controlling the filament’s self-rupture time, gradual facilitation of the conductance can be achieved. Moreover, various device engineering methods also offer opportunities to achieve analog switching in CBRAM devices. Furthermore, because of their volatile TS features, CBRAMS are extremely effective as artificial neurons. The LIF artificial neurons have been demonstrated with various TS CBRAM devices. In addition, the possibility of implementing single device artificial neurons with CBRAMs offers opportunities for high-scale integration. CBRAMs also offer great opportunities for other emerging applications, such as the implementation of a true random number generator (TRNG) and physical unclonable function (PUF) [85,86].

## Figures and Tables

**Table 1:** Summary of key memory characteristics reported in several CBRAM devices based on different electrode and switching materials.

Device Structure	V_SET_/V_RESET_ (V)	ON/OFF Ratio	Endurance (Cycles)	Retention (s)	Refs.
Ag/HfO_2_/Pt	+0.19/−0.23	10^5^	10^4^	5 × 10^4^	[17]
Ag/HfO_2_/Pt	+0.6/−0.5	10^9^	10^8^	-	[53]
AgTe/HfO_2_/Pt	-	10^9^	10^9^	-	[53]
Ag/ZrO_2_/MoS_2_/Pt	+0.8/−0.2	10^6^	10^2^	10^4^	[54]
Ag/Ta_2_O_5_/Pt	+0.15/−0.5	10^4^	>450	>10^4^	[18]
Cu/Al_2_O_3_/TiN	+2/−1	10^4^	-	-	[36]
Co/Al_2_O_3_/TiN	+2/−1	10^4^	10^7^	5 × 10^4^	[36]
Co/LaSiO/TiN	+1.2/−0.5	600	>10^3^	-	[36]
Sn/HfO_2_/Sn/Pt	+3/−2	10^4^	225	10^5^	[37]
Cu/Ta_2_O_5_/TaO_x_/Ta/W	+2/−1	10^3^	>10^6^	>10^4^	[43]
Cu/SiO_2_/Pt	+0.75/−0.64	~10	-	-	[55]
Ag/SiO_2_/Pt	+0.86/−0.67	10^4^	-	-	[55]
Te/MgO/HfO_x_/TiN	+0.7/−0.8	~10	10^4^	10^4^	[35]
Cr-Au/InSe/Ag	+1/−1	10^2^	>50	>10^5^	[50]
Ag/GeSe/Pt	+0.2/−0.2	10^3^	>10^6^	>10^5^	[23]
Cu/BP/Au	+0.6/−0.6	10^5^	25	10^4^	[25]
Cu/GST/Pt	+0.5/−0.5	10^4^	-	10^4^	[56]
Cu/GeTe/TiN	+0.75/−0.75	10^4^	>10^4^	>10^4^	[21]

## Data Availability

Not applicable.
